# The effect of uniaxial strain on graphene nanoribbon carrier statistic

**DOI:** 10.1186/1556-276X-8-479

**Published:** 2013-11-14

**Authors:** Zaharah Johari, Razali Ismail

**Affiliations:** 1 Faculty of Electrical Engineering, Universiti Teknologi Malaysia, Johor Bahru, Johor 81310, Malaysia

**Keywords:** GNR, Uniaxial strain, Carrier statistic

## Abstract

Armchair graphene nanoribbon (AGNR) for *n*=3*m* and *n*=3*m*+1 family carrier statistic under uniaxial strain is studied by means of an analytical model based on tight binding approximation. The uniaxial strain of AGNR carrier statistic models includes the density of state, carrier concentration, and carrier velocity. From the simulation, it is found that AGNR carrier concentration has not been influenced by the uniaxial strain at low normalized Fermi energy for *n*=3*m* and *n*=3*m*+1. In addition, the carrier velocity of AGNR is mostly affected by strain at high concentration of *n*≈3.0×10^7^ and 1.0 × 10^7^ m^−1^ for *n*=3*m* and *n*=3*m*+1, respectively. The result obtained gives physical insight into the understanding of uniaxial strain in AGNR.

## Background

Graphene has attracted numerous research attention since it was isolated in 2004 by Novoselov et al. [1]. Due to its unique hexagonal symmetry, graphene posses many remarkable electrical and physical properties desirable in electronic devices. It is the nature of graphene that it does not have a bandgap, which has limited its usage. Therefore, efforts to open up a bandgap has been done by several methods [2-4]. The most widely implemented method is patterning the graphene into a narrow ribbon called graphene nanoribbon (GNR) [4]. Recently, strain engineering have started to emerge in graphene electronics [5]. It is found that strain applied to graphene can modify its band structure, thus, altering its electronic properties [6-8]. In fact, uniaxial strain also helps in improving the graphene device’s electrical performance [9]. Similar characteristics have been observed when strain is applied to conventional materials like silicon (Si), germanium (Ge), and silicon germanium (SiGe) [10]. Strain in graphene can be characterized by two major varieties, namely uniaxial and shear. This strain behaves differently on graphene depending on the edge shape, namely zigzag or armchair [8]. The presence of the strain effect in graphene is by the *G* peak that splits and shifts in the Raman spectrum [11,12]. It is worth noting that strain in graphene may unintentionally be induced during the fabrication of graphene devices.

Computational modeling and simulation study pertaining to strain graphene and GNR for both the physical and electrical properties have been done using few approaches such as the tight binding model and the ab initio calculation [6,13]. An analytical modeling approach has also been implemented to investigate the strain effect on GNR around the low-energy limit region [14,15]. However, most of the previous works have only focused on the electronic band structure, particularly the bandgap. As the carrier transport in GNR has a strong relation with this electronic band structure and bandgap, it is mandatory to investigate the strain effect on the carrier transport such as carrier density and velocity. Therefore, in this paper, an analytical model representing uniaxial strain GNR carrier statistic is derived based on the energy band structure established by Mei et al. [15]. The strain effect in our model is limited to low strain, and only the first subband of the AGNR *n*=3*m* and *n*=3*m*+1 families is considered. In the following section, the analytical modeling of the uniaxial strain AGNR model is presented.

## Methods

### Uniaxial strain AGNR model

The energy dispersion relation of GNR under tight binding (TB) approximation incorporating uniaxial strain is represented by Equation 1 taken from reference [15]. The TB approximation is found to be sufficient in the investigation for small uniaxial strain strength. This is because the state near the Fermi level is still determined by the 2*p*_*z*_ orbitals that form the *π* bands when the lattice constant changes [6]:

(1) $$\begin{array}{c} \vec{E(n,k)}\;=\pm \left[\right)t_{1}^{2}+4t_{2}^{2}\overset{2}{\cos}\left(\frac{\mathrm{p\pi}}{n+1}\right)\\ \;+4t_{1}t_{2}\cos\left(\frac{\mathrm{p\pi}}{n+1}\right)\cos\left(\frac{3}{2}k_{x}a\right){])}^{1/2} \end{array}$$

where $t_{1}=\frac{t_{0}}{{\left(1+\varepsilon \right)}^{2}}$, $t_{2}=t_{3}=\frac{t_{0}}{\left(1+\frac{\varepsilon }{4}\right)}$, *t*_0_=−2.74 eV is the unstrained hopping parameter, *a*=0.142 nm is the lattice constant and *t*_1_ and *t*_2_ are the deformed lattice vector hopping parameter of the strained AGNR. *ε* is the uniaxial strain [15].

Using the first-order trigonometric function, Equation 1 can further be simplified to the following equation:

(2) $$\begin{array}{c} \vec{E(n,k)}\;=\pm \left[\right)t_{1}^{2}+4t_{2}^{2}\overset{2}{\cos}\left(\frac{\mathrm{p\pi}}{n+1}\right)\\ \;+4t_{1}t_{2}\cos\left(\frac{\mathrm{p\pi}}{n+1}\right)\left(1-\frac{9}{8}a^{2}k_{x}^{2}\right){])}^{1/2} \end{array}$$

To model the bandgap, at *k*_*x*_=0, Equation 2 is reduced to [15]

(3) $$E_{c}=E_{v}=E\left(k_{x}=0\right)=\left|t_{1}+2t_{2}\cos\left(\frac{\mathrm{p\pi}}{n+1}\right)\right|$$

Thus, the bandgap is obtained as the following equation [15]:

(4) $$E_{g}=2E\left(k_{x}=0\right)=2\left|t_{1}+2t_{2}\cos\left(\frac{\mathrm{p\pi}}{n+1}\right)\right|$$

The energy dispersion relation from Equation 2 can further be simplified to

(5) $$\vec{E(n,k)}=\pm \sqrt{{\left(\frac{E_{g}}{2}\right)}^{2}+Bk_{x}^{2}}$$

where

(6) $$B=-\frac{9}{2}t_{1}t_{2}\cos\left(\frac{\mathrm{p\pi}}{n+1}\right)a^{2}$$

Equation 5 will be the basis in the modeling of strain GNR carrier statistic. GNR density of state (DOS) is further derived. The DOS that determines the number of carriers that can be occupied in a state of the system [16] is yielded as in Equation 7:

(7) $$\text{DOS}(E\;)=\frac{1}{2\pi \sqrt{B}}\frac{E}{\sqrt{E^{2}-{\left(\frac{E_{g}}{2}\right)}^{2}}}$$

In the modeling of the strain GNR carrier concentration, energy dispersion relation is approximated with the parabolic relation, $\sqrt{1+\alpha^{2}}\approx +\frac{1}{2}\alpha$. By substituting the normalized Fermi energy as $\eta =\frac{E_{F}-E_{g}/2}{k_{B}T}$, the strain AGNR carrier concentration model is derived and represented by

(8) $$n=\frac{\sqrt{k_{B}T}\sqrt{E_{g}}}{4\sqrt{B}\sqrt{\pi }}I_{-\frac{1}{2}}(\eta )$$

To further evaluate the intrinsic carrier velocity in response to the uniaxial strain, the following definition is referenced [17]:

(9) $$v_{i}=\frac{v_{f}\int \text{DOS}(E\;)\times f(E\;)}{\text{DOS}(E\;)\times f(E\;)}\text{dE}$$

The Fermi velocity, *v*_*f*_, is modeled as in reference [18]. Thus, *v*_*f*_ is obtained as the following equation:

(10) $$v_{f}=\frac{\sqrt{B}}{\hbar }\frac{\sqrt{E^{2}-{\left(\frac{E_{g}}{2}\right)}^{2}}}{E}$$

Hence, using the intrinsic velocity model defined in Equation 9, the strain AGNR intrinsic carrier velocity yields the following equation:

(11) $$v_{i}=\frac{2\pi \sqrt{B}\sqrt{k_{B}T}}{\hbar \sqrt{E_{g}}}\frac{I_{0}(\eta )}{I_{-1/2}(\eta )}$$

The analytical model presented in this section is plotted and discussed in the following section.

## Results and discussion

**Figure 1:**
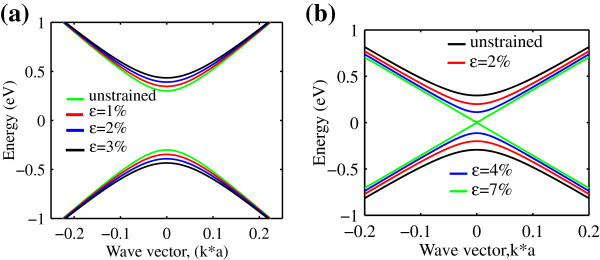
**Energy band structure of uniaxial strain AGNR (a)*n=3m* and (b)*n=3m+1* for the model in Equation** 1.

The energy band structure in respond to the Bloch wave vector, *k*_*x*_, modeled as in Equation 1 which was established by Mei et al. [15], is plotted in Figure 1 for *n*=3*m* and *n*=3*m*+1 family, respectively. For each simulation, only low strain is tested since it is possible to obtain experimentally [12]. It can be observed from both figures that there is a distinct behavior between the two families. For *n*=3*m*, the separation between the conduction and valence bands, which is also known as bandgap, increases with the increment of uniaxial strain. On the contrary, the *n*=3*m*+1 family exhibits decrements in the separation of the two bands. It is worth noting that the *n*=3*m*+1 family also shows a phase metal-semiconductor transition where at 7% of strain strength, the separation of the conduction and valence bands almost crosses at the Dirac point. This is not observed in the *n*=3*m* family [15].

The hopping integral *t*_0_ between the *π* orbitals of AGNR is altered upon strain. This causes the up and down shift, the *σ*^∗^ band, to the Fermi level, *E*_*F*_[19]. These two phenomena are responsible for the bandgap variation. It has been demonstrated that GNR bandgap effect with strain is in a zigzag pattern [14]. This observation can be understood by the shifting of the Dirac point perpendicular to the allowed *k* lines in the graphene band structure and makes some bands closer to the Fermi level [7,8]. Hence, the energy gap reaches its maximum when the Dirac point lies in between the two neighboring *k* lines. The allowed *k* lines of the two families of the AGNR have different crossing situations at the *K* point [8]. This may explain the different behaviors observed between *n*=3*m* and *n*=3*m*+1 family.

**Figure 2:**
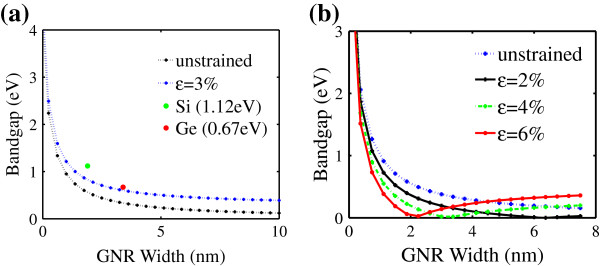
**Bandgap of AGNR in respond to the width for (a)** ***n=3m* and (b)** ***n=3m+1*.**

To further evaluate, the GNR bandgap versus the GNR width is plotted in Figure 2. Within the uniaxial strain strength investigated, the bandgap of the *n*=3*m* family is inversely proportional to the GNR width. The narrow bandgap at the wider GNR width is due to the weaker confinement [20]. The conventional material of Si and Ge bandgaps are also plotted in Figure 2 for comparison. In order to achieve the amount of bandgap similar to that of Si (1.12 eV) or Ge (0.67 eV), the uniaxial strain is projected to approximately 3% for the *n*=3*m* family. A similar observation can be seen for *n*=3*m*+1 with 2% uniaxial strain. However, a higher strain resulted in a different kind of observation. For example at 4% uniaxial strain, the phase transition from metallic to semiconductor occurs at a GNR width of approximately 3*m*. The phase transition is not observed in AGNR *n*=3*m*[15]. When higher strain is applied, the phase transition occurs at a lower width. The difference in GNR width for the phase transition to occur depends on the subband spacing effect with GNR width [21]. The constitution of the phase transition suggests that the GNR bandgap can be tuned continuously between the metal and semiconductor by applying strain.

**Figure 3:**
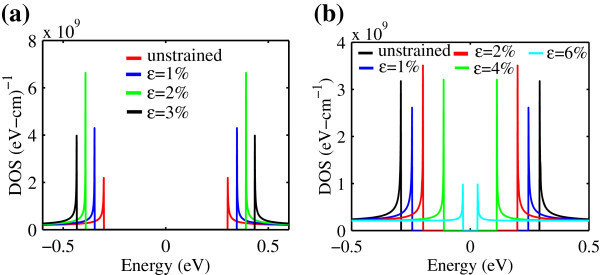
**DOS varying the uniaxial strain strength in AGNR (a)** ***n=3m* and (b)** ***n=3m+1*.**

Based on the energy band structure, the analytical model representing the DOS of strained AGNR is derived as in Equation 7. It is necessary to understand the DOS of strain AGNR as it will give insight on the amount of carriers that can be occupied in a state. The analytical model for strained AGNR is shown in Figure 3 for the first subband for the two AGNR families. It appears that the patterns of DOS are essentially the same for both AGNR families. It can be observed from Figure 3a,b that the Van Hove singularities are present at the band edge. For AGNR with *n*=3*m*, the increment of strain increases the DOS remarkably. However, when *ε*=3*%*, despite the wide bandgap, the DOS substantially decreases. This is the reason for changing the band index, *p*, which corresponds to the bandgap [15]. In the case of *n*=3*m*+1, the DOS exhibits the opposite. In fact, when the strain strength made the band approach the transition phase, the DOS reduces significantly; at the same time, the bandgap approaches zero.

**Figure 4:**
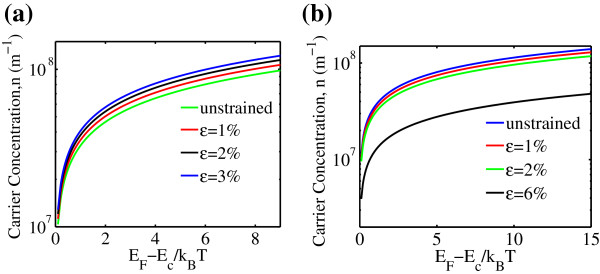
**Uniaxial strained AGNR carrier concentration as a function of normalized Fermi energy for (a)** ***n=3m* and (b)** ***n=3m+1*.**

To assess the effect of strain on AGNR carrier concentration, the computed model as in Equation 8 as a function of *η* is shown in Figure 4. Apparently, the amount of carriers increases when the AGNR *n*=3*m* is added with uniaxial strain. Conversely, AGNR *n*=3*m*+1 shows a reduction in carrier concentration upon strain. Most notably, for AGNR *n*=3*m*, the carrier concentration converges at low *η* within the investigated strain level. Meanwhile, the carrier concentration exhibits considerable effect upon the strain when the Fermi level lies at 3 *k*_*B*_T away from the conduction or valence band edge. The same observation was achieve in AGNR *n*=3*m*+1.

**Figure 5:**
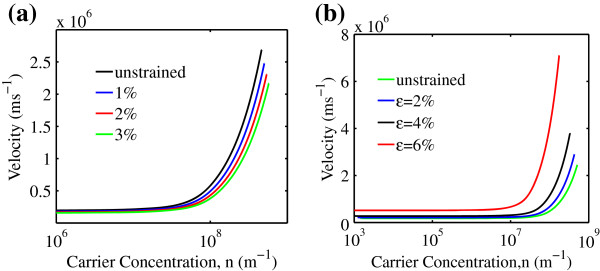
**Uniaxial strained AGNR carrier velocity in response to carrier concentration for (a)** ***n=3m* and (b)** ***n=3m+1*.**

To assess the carrier velocity effect with carrier concentration upon the strained AGNR, the analytical model in Equation 10 is plotted in Figure 5. It can be seen from Figure 5a,b that the GNR carrier velocity decreases and increases with the applied uniaxial strain for AGNR *n*=3*m* and AGNR *n*=3*m*+1 families, respectively. Inspection of these figures also showed that the uniaxial strain mostly affected the carriers at high concentration. This is evident by the curves that tend to converge until *n*≈3×10^7^m^−1^ and has an almost constant velocity at 1.8 × 10^5^ ms ^−1^. When the concentration is high enough, the uniaxial strain starts to give a considerable effect to the velocity. This is supported by the previous observation in Figure 4 where the effect of the strain is infinitesimal at low *η*. In fact, the applied strain also affects the degeneracy approach. The strained AGNR *n*=3*m* approach degenerated later compared to the unstrained AGNR. A similar behavior was also observed in the AGNR *n*=3*m* + 1 family except that strained AGNR approaches degeneracy faster compared to their unstrained counterparts. This indicates that uniaxial strain is beneficial at a high concentration regime. Nonetheless, this is not unreasonable for low-dimensional nanostructures like GNR since it is mostly in the degenerated realm particularly for narrow width.

**Figure 6:**
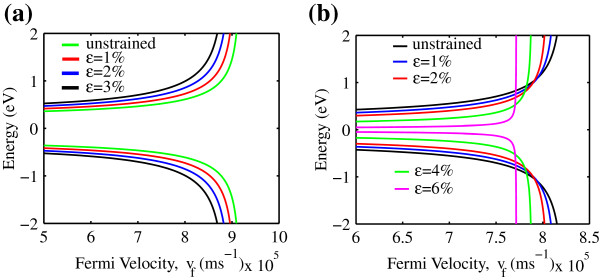
**Fermi velocity effect to the energy band structure of uniaxial strain AGNR for (a)** ***n=3m* and (b)** ***n=3m+1*.**

The energy in response to the Fermi velocity of strained AGNR is shown in Figure 6. It can be observed that the effect of the strain on the Fermi velocity for both AGNR families is dramatic. Both AGNR *n*=3*m* and *n*=3*m*+1 have appreciable reduction in the Fermi velocity when the uniaxial strain increases as can be seen in Figure 6a,b. This reduction is attributed to the decrements in the *π* orbital overlap [22] in the AGNR band structure. As a consequence, the mobility is predicted to be degraded [23] as a result of the strong effect in the interaction of the strained carbon atoms [18,23].

## Conclusions

In this paper, the uniaxial strain AGNR for *n*=3*m* and *n*=3*m* + 1 family carrier statistic is analytically modeled, and their behaviors are studied. It is found that uniaxial strain gives a substantial effect to AGNR carrier statistic within the two AGNR families. The AGNR carrier concentration has not been influenced by the uniaxial strain at low normalized Fermi energy. It is also shown that the uniaxial strain mostly affects carrier velocity at a high concentration of *n*≈3.0×10^7^ m ^−1^ and *n*≈1.0×10^7^ m ^−1^ for *n*=3*m* and *n*=3*m*+1, respectively. In addition, the Fermi velocity of the AGNR *n*=3*m* and *n*=3*m*+1 exhibits decrements upon the strain. Results obtained give physical insight on the understanding of the uniaxial strain effect on AGNR. The developed model in this paper representing uniaxial strain AGNR carrier statistic can be used to further derive the current-voltage characteristic. This computational work will stimulate experimental efforts to confirm the finding.

## Competing interests

The authors declare that they have no competing interests.

## Authors’ contributions

ZJ carried out the analytical modelling and simulation studies. RI participated in drafting and improving the manuscript. Both authors read and approved the final manuscript.

## References

[B1] Novoselov KS Geim AK Morozov SV Jiang D Zhang Y Dubonos SV Grigorieva IV Firsov AA Electric field effect in atomically thin carbon films Science 2004 8 5696 666 669 10.1126/science.1102896 15499015

[B2] Castro EV Novoselov KS Morozov SV Peres NMR dos Santos JMBL Nilsson J Guinea F Geim AK Neto AHC Biased bilayer graphene: semiconductor with a gap tunable by the electric field effect Phys Rev Lett 2007 8 216802 18233240 10.1103/PhysRevLett.99.216802

[B3] Nourbakhsh A Cantoro M Vosch T Pourtois G Clemente F van der Veen MH Hofkens J Heyns MM Gendt SD Sels BF Bandgap opening in oxygen plasma-treated graphene Nanotechnology 2010 8 43 435203 10.1088/0957-4484/21/43/435203 20890016

[B4] Li X Wang X Zhang L Lee S Dai H Chemically derived, ultrasmooth graphene nanoribbon semiconductors Science 2008 8 1229 1232 10.1126/science.1150878 18218865

[B5] Pereira VM Neto AHC Strain engineering of graphene’s electronic structure Phys Rev Lett 2009 8 4 046 801+ 10.1103/PhysRevLett.103.046801 19659379

[B6] Gui G Li J Zhong J Band structure engineering of graphene by strain: first-principles calculations Phys Rev B 2008 8 7 075435

[B7] Rosenkranz N Mohr M Thomsen C Uniaxial strain in graphene and armchair graphene nanoribbons: an ab initio study Annalen der Physik 2011 8 1-2 137 144 10.1002/andp.201000092

[B8] Li Y Jiang X Liu Z Liu Z Strain effects in graphene and graphene nanoribbons: the underlying mechanism Nano Res 2010 8 8 545 556 10.1007/s12274-010-0015-7

[B9] Alam K Uniaxial strain effects on the performance of a ballistic top gate graphene nanoribbon on insulator transistor Nanotechnol IEEE Trans 2009 8 4 528 534

[B10] Lee ML Fitzgerald EA Bulsara MT Currie MT Lochtefeld A Strained Si, SiGe, and Ge channels for high-mobility metal-oxide-semiconductor field-effect transistors J Appl Phys 2005 8 1 011101 10.1063/1.1819976

[B11] Mohiuddin TMG Lombardo A Nair RR Bonetti A Savini G Jalil R Bonini N Basko DM Galiotis C Marzari N Novoselov KS Geim AK Ferrari AC Uniaxial strain in graphene by Raman spectroscopy: g peak splitting, Grüneisen parameters, and sample orientation Phys Rev B 2009 8 205433

[B12] Ni ZH Yu T Lu YH Wang YY Feng YP Shen ZX Uniaxial strain on graphene: Raman spectroscopy study and band-gap opening ACS Nano 2008 8 11 2301 2305 10.1021/nn800459e 19206396

[B13] Mohr M Papagelis K Maultzsch J Thomsen C Two-dimensional electronic and vibrational band structure of uniaxially strained graphene from ab initio calculations Phys Rev B 2009 8 205410

[B14] Lu Y Guo J Band gap of strained graphene nanoribbons Nano Res 2010 8 3 189 199 10.1007/s12274-010-1022-4

[B15] Mei H Yong Z Hong-Bo Z Effect of uniaxial strain on band gap of armchair-edge graphene nanoribbons Chin Phys Lett 2010 8 3 037302 10.1088/0256-307X/27/3/037302

[B16] Datta S Quantum Transport : Atom to Transistor 2005 Cambridge: Cambridge University Press

[B17] Ahmadi MT Ismail R Tan MLP Arora VK The ultimate ballistic drift velocity in carbon nanotubes J Nanomaterials 2008 8 2008 769250

[B18] Wong J-H Wu B-R Lin M-F Strain effect on the electronic properties of single layer and bilayer graphene J Phys Chem C 2012 8 14 8271 8277 10.1021/jp300840k

[B19] Liao WH Zhou BH Wang HY Zhou GH Electronic structures for armchair-edge graphene nanoribbons under a small uniaxial strain Eur Phys J B 2010 8 463 467 10.1140/epjb/e2010-00222-3

[B20] Sun L Li Q Ren H Su H Shi QW Yang J Strain effect on electronic structures of graphene nanoribbons: A first-principles study J Chem Phys 2008 8 7 074704 10.1063/1.2958285 19044789

[B21] Chang CP Wu BR Chen RB Lin MF Deformation effect on electronic and optical properties of nanographite ribbons J Appl Phys 2007 8 6 063506 10.1063/1.2710761

[B22] Huang M Yan H Heinz TF Hone J Probing strain-induced electronic structure change in graphene by raman spectroscopy Nano Lett 2010 8 10 4074 4079 10.1021/nl102123c 20735024

[B23] Shah R Mohiuddin TMG Singh RN Giant reduction of charge carrier mobility in strained graphene Mod Phys Lett B 2013 8 03 1350021 10.1142/S0217984913500218

